# Impacts of autochthonous particulate organic matter on redox-conditions and elimination of trace organic chemicals in managed aquifer recharge

**DOI:** 10.1007/s11356-023-25286-0

**Published:** 2023-01-23

**Authors:** Josefine Filter, Till Ermisch, Aki Sebastian Ruhl, Martin Jekel

**Affiliations:** 1 Technische Universität Berlin, Chair of Water Quality Control, KF4, Straße des 17. Juni 135, 10623 Berlin, Germany; 2 German Environment Agency, Section II 3.3, Schichauweg 58, 12307 Berlin, Germany

**Keywords:** Soil aquifer treatment, Algae, Trace organic chemicals, Particulate organic matter

## Abstract

Autochthonous carbon fixation by algae and subsequent deposition of particulate organic matter can have significant effects on redox conditions and elimination of trace organic chemicals (TOrCs) in managed aquifer recharge (MAR). This study investigated the impacts of different algae loadings (0–160 g/m^2^) and infiltration rates (0.06–0.37 m/d) on overall oxygen consumption and elimination of selected TOrCs (diclofenac, formylaminoantipyrine, gabapentin, and sulfamethoxazole) in adapted laboratory sand columns. An infiltration rate of 0.37 m/d in conjunction with an algae load of 80 g/m^2^ (dry weight) sustained oxic conditions in the sand bed and did not affect the degradation of TOrCs. Thus, the availability of easily degradable organic carbon from algae did not influence the removal of TOrCs at an influent concentration of 1 µg/L. In contrast, a lower infiltration rate of 0.20 m/d in combination with a higher algae loading of 160 g/m^2^ caused anoxic conditions for 30 days and significantly impeded the degradation of formylaminoantipyrine, gabapentin, sulfamethoxazole, and diclofenac. Especially the elimination of gabapentin did not fully recover within 130 days after pulsed algae deposition. Hence, measures like micro-sieving or nutrient control are required at bank filtration or soil aquifer treatment sites with low infiltration rates.

**Supplementary information:**

The online version contains supplementary material available at 10.1007/s11356-023-25286-0.

## Introduction

In partially closed water cycles, surface waters used for drinking water supply can contain significant amounts of treated wastewater (Karakurt et al. 2019). This challenges managed aquifer recharge (MAR) systems such as soil aquifer treatment (SAT) and bank filtration (BF), which are close-to-nature and cost-efficient treatments for water reclamation and provision of raw water for drinking water supply. In SAT, surface water is infiltrated in artificial basins and regained by nearby wells, whereas in BF, groundwater is captured from a well near or under a river or lake to induce infiltration from the surface water body through a soil passage (Dillon 2005). In the aquifer and infiltration zone, the water is exposed to various physico-chemical and biological processes that eliminate pathogens, dissolved organic matter (DOM), particulate organic matter (POM), and many trace organic chemicals (TOrCs) including pharmaceutically active compounds, personal care products, biocides, and industrial chemicals (Betancourt et al. 2014; Grünheid et al. 2005). These contaminants, also referred to as contaminants of emerging concern (CECs) or organic micropollutants (OMP) in literature, can be found in ng/L to µg/L in surface- and groundwater. Therefore, the German Environment Agency set human health threshold values (GOW) for drinking water for a growing number of substances including the antiepileptic gabapentin (1 µg/L), the anti-inflammatory drug diclofenac (0.3 µg/L), and formylaminoantipyrine, a transformation product of the anti-inflammatory drug metamizol (0.3 µg/L) (UBA 2019).

The efficiency of TOrCs degradation in MAR depends on various factors such as microbial colonization, temperature, redox conditions, and infiltration rate. An improved elimination of moderately degradable TOrCs was found under oxic and carbon limited conditions provoked by a hardly biodegradable DOC in field studies by Hellauer et al. (2017a) and Regnery et al. (2015). Carbon limited conditions are referred to in literature, if the concentration of biodegradable carbon is below 1 mg/L (Hoppe-Jones et al. 2012; Regnery et al. 2016) or even below 0.7 mg/L (Hellauer et al. 2019). Beside the redox conditions, the availability of BDOC was reported to influence the microbial community composition and diversity in MAR systems (Li et al. 2012).

The proportion of endogenous, autochthonous carbon strongly depends on location-based factors like nutrient availability or temperature, morphology of the surface water. However, studies investigating POM sources mostly only distinguish between endogenous POM and terrestrial POM, e.g., by stable C-N isotope analysis (Meng et al. 2021). Typically, SAT infiltration basins and surface waters at BF sites are exposed to light and thus provoke algae growth. Wilt et al. (2016) investigated TOrCs removal in an algae reactor and observed a removal of several TOrCs. However, as for diclofenac, the enhanced removal was mainly attributed to photolysis rather than biodegradation. After infiltration in BF and SAT, the deposited autochthonous algae can contribute to the formation of a cake layer, the so-called “Schmutzdecke,” and cause a decline in infiltration rates due to clogging (Hoffmann and Gunkel 2011). Even if the organic content of the influent water is low, algal carbon fixation in biomass can introduce new organic carbon into the sand filter systems. The molar Redfield ratio for phytoplankton composition (in marine environments) of 106:16:1 for C:N:P (Redfield, 1934) indicates the potential of C fixation: A total phosphor concentration of e.g. 0.1 mg/L could lead to a theoretical carbon accumulation of 4.2 mgC/L. The degradation of the filtrated dead algae can cause a severe consumption of dissolved oxygen and thus an oxygen limitation in the subsequent sand layer. The possible change of redox conditions from oxic to anoxic (nitrate reducing) or even anaerobic (sulfate reducing) affects especially the first decimeters of the infiltration zone, which is considered as highly biologically active (Essandoh et al. 2011; Gross-Wittke et al. 2010). Additionally, the release of organic carbon could serve as additional carbon source, outcompeting direct degradation of TOrCs. There have been various studies investigating DOC removal and its impact on TOrCs elimination (Amy and Drewes 2007; Grünheid et al. 2005), but there is only little information on the effects of particulate organic matter on TOrCs removal in MAR systems.

Column studies have investigated the influence of POM in sediments and in form of leave litter deposition on redox conditions and TOrC removal in natural sand filter systems (Bayarsaikhan et al. 2018; Filter et al. 2017). Accumulated algae biomass managed aquifer recharge was mainly considered to describe the formation of the clogging layer. However, the effect of this autochthonous POM on the oxygen regime and TOrC removal is hardly investigated.

Therefore, the aim of the present study was to investigate the effects of (i) different algae loads and (ii) different infiltration rates on TOrCs removal (1 µg/L initial TOrCs concentration). Two experimental series with four columns each were operated for experiments with variations of algae load (series A) and infiltration rate variations (series B). Besides oxygen consumption and DOC release, the analgesic diclofenac, the antibiotic sulfamethoxazole, the anticonvulsants gabapentin and carbamazepine, and the corrosion inhibitor benzotriazole as well as formylaminoantipyrine were monitored. The selection of target TOrCs is based on data from previous studies (Burke et al. 2013; Filter et al. 2017; Hellauer et al. 2017b) and suggestions for process indicator substances (Jekel et al. 2015). We hypothesize that even low amounts of algae affect TOrCs degradation due to an increase of BDOC as primary substrate, even though oxic conditions are preserved. If algae loadings are so severe that anoxic conditions occur, high infiltration rates might help to re-install favorable oxic conditions to recover the microbial elimination of certain TOrCs.

## Materials and methods

### Algae enrichment

Indigenous microalgae from Lake Tegel water were cultivated in a 10 L growth reactor with a fertilizer mix (Seramis GmbH, Germany), CO_2_ aeration once a day for 5 minutes and light exposure for 18 h using fluorescent lamps. The reactor was stirred with 80 rpm for 10 minutes every hour. Microscopic observation revealed the enrichment of coccal green algae. Intermittent stirring was stopped after 2 months and the settled algae (after 24 h sedimentation) were washed three times with tap water and further concentrated by centrifugation for five minutes at 2,000 rpm. After the last centrifugation step, the algae were suspended in 0.25 L tap water and the dry weight (total suspended solids (TSS)) of the suspension was determined by membrane filtration with 0.45 µm and quantification of the weight increase of the dried filter.

### Long-term column experiments

Acrylic-glass columns (70 mm (series A) and 65 mm (series B) inner diameters) were filled to a height of 55 mm with supporting gravel at the bottom and additional 300 mm sand (effective particle diameter: 0.2 mm) from an groundwater recharge basin, excluding the colmation layer (Filter et al. 2017). The water levels of the supernatants (open to the atmosphere) were adjusted by the position of the effluent outlet. The systems were operated at 20–25 °C and without daylight to avoid phototrophic activity. Samples for further TOC, DOC, and TOrC analysis were taken from the influent tank and by collecting the effluents in 1 L glass bottles (as 20 h composite samples). All columns were fed with (non-chlorinated) Berlin tap water originating from BF. Due to the soil passage of more than 50 days in the respective water supply system, the influent concentration of dissolved organic carbon (DOC) of 4–5 mg/L consists mainly of refractory humic substances. To enrich the influent with TOrCs, defined volumes of a stock solution were added to the tap water to adjust TOrCs concentrations of 1 µg/L each. The stock solution consisted of 10 mg/L of benzotriazole, diclofenac, carbamazepine, formylaminoantipyrine, gabapentin, and sulfamethoxazole dissolved in ultrapure water. The influent tank was aerated with a porous air stone to maintain oxygen saturated conditions and had to be refilled regularly with the spiked tap water.

After an initial phase of 179 days for series A and 256 days for series B, the algae were deposited on the sand by circulating the algae suspensions (200 mL) during 20 h through the sand column with an elevated volume flow of 250 mL/h to avoid sedimentation within tubes.

The four columns of series A received increasing algae loads of 0, 20, 40, and 80 g/m^2^ (according to dry weights). They were operated with an average infiltration rate of 0.38 ± 0.02 m/d (m^3^ per m^2^ filter area and day), resulting in a hydraulic retention time (HRT) of ca. 10 h (calculation of the HRT included in the supplementary material section 2). The biomass of 80 g/m^2^ could be accumulated in a recharge basin operated for approximately 123 days at an infiltration rate of 0.4 m/d with a mesotrophic/eutrophic raw water assuming a phosphorous concentration of 0.02 mg/L (detailed calculations provided in the supplementary material section 1). Hoffmann and Gunkel (2011) calculated 20 to 50 g/m^2^ as POM input into sediments of a mesotrophic lake. In this way, series A represents reasonable algae amounts, which can be accumulated within less than a year. After the columns of series A stabilized, the infiltration velocity of series A was decreased about 30% to 0.29 ± 0.06 m/d from day 56 onwards. In this way, we wanted to capture possible long-term but less pronounced effects of POM accumulation.

**Fig. 1:**
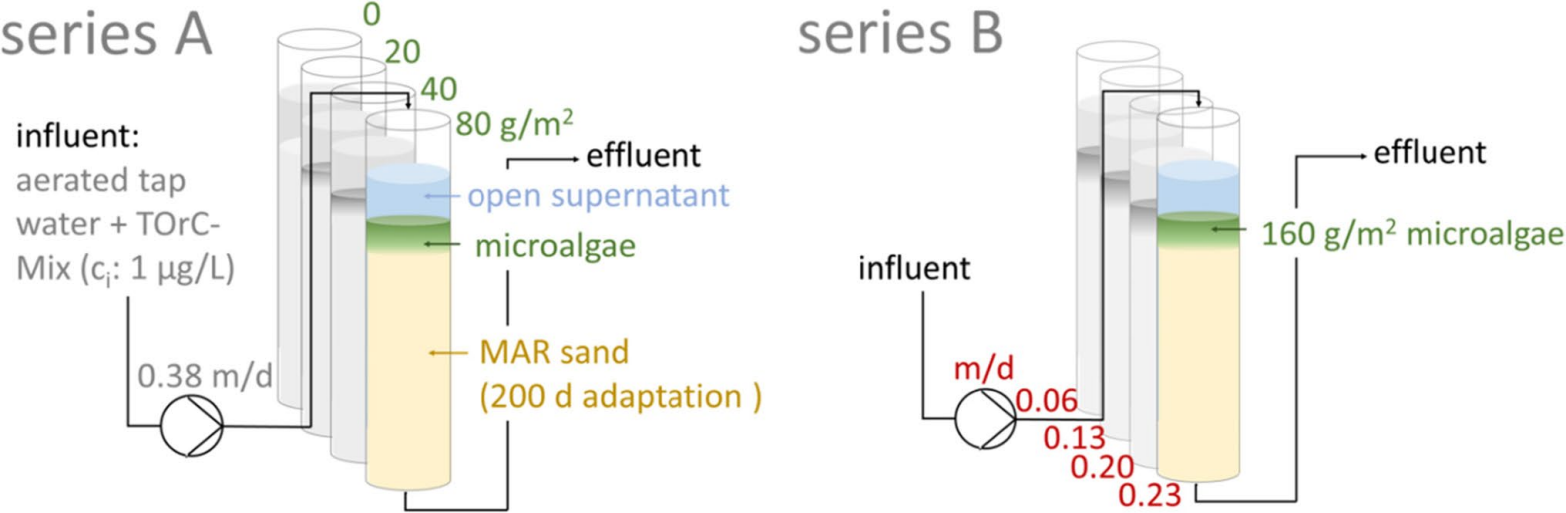
Set-up of the column systems with varying algae amounts (series A) and varying infiltration rates (series B)

To provoke stronger effects on redox conditions and TOrCs removal, the four columns of series B were operated at high algae loadings of 160 g/m^2^. To investigate the impact of the filter velocity on such a system, the infiltration rates (±standard deviation) were varied between 0.06 (±0.00), 0.13 (±0.01), 0.20 (±0.01), and 0.23 (±0.05) m/d. Hoffmann and Gunkel (2011) measured infiltration rates between 0.02 and 0.65 m/d during BF. In a technical slow sand filter, the designed rate of downward flow under optimum conditions is reported to range between 2.4 and 9.6 m/d (Huisman and Wood 1974). Figure 1 schemes the set-up of the two column systems.

### Analyses

Dissolved oxygen was measured with optodes (PreSens Precision Sensing, Germany), which were installed as flow through cells in the influent and effluent tubes. Except for total organic carbon (TOC) analyses, all samples were vacuum-filtered with 0.45 µm cellulose nitrate membranes (Sartorius, Germany). The TOC and DOC were quantified with a TOC Cube analyzer (Elementar, Germany) and further characterized with liquid chromatography and continuous organic carbon detection (LC-OCD, DOC-Labor Huber, Germany) (Huber et al. 2011). For quantifying the amount of double bonds and aromatic groups of the DOM, ultraviolet light (UV) absorption at 254 nm wavelength was measured with the spectral photometer Lambda 12 (Perkin Elmer, USA) using a cuvette of 1 cm optical length. The selected TOrCs were quantified by high performance liquid chromatography coupled with tandem mass spectrometry (HPLC-MS/MS). Besides calibrating TOrC concentration with nine standards, deuterated internal standards of 1 µg/L were used to comply with matrix effects. The method is described in detail by Hellauer et al. (2017b).

## Results and discussion

### Impact of algae mass on oxygen consumption

**Fig. 2:**
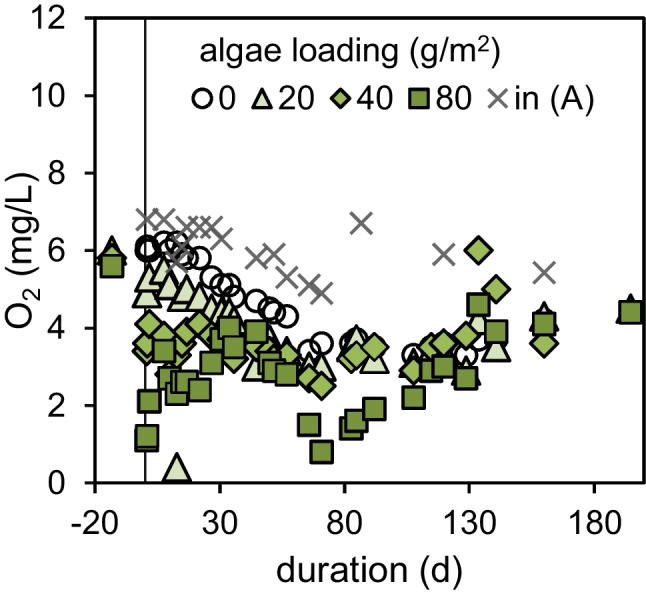
Influent (in (A)) and effluent concentrations of dissolved oxygen (O_2_) in columns of series A with different algae loadings (expressed as g/m^2^ TSS) introduced at day 0 and an average filtration rate of 0.38 m/d. (Please note that after day 56 the filtration rate was decreased to 0.29 m/d, provoking a decrease in effluent oxygen concentration)

The oxygen consumption increased in all sand columns with the addition of algae for 70 days (Fig. 2). Algae amounts of 80 g/m^2^ or less did not cause anoxic conditions in the following sand layer and an average infiltration rate of 0.38 m/d is retained.

The reference column only filled with sand indicated an average background oxygen consumption of 1–2 mg/L caused by accumulated organic matter in the sand, which was also found in previous studies (Filter et al. 2017).

The applied algae mass contained 0.5 g C/g TSS. Assuming a specific oxygen demand of 2.7 g molecular oxygen for the complete mineralization of 1 g organic carbon with an average oxidation number of ±0 (M_O2_/M_C_), the potential oxygen consumption can be calculated per gram dry matter. Further details for this calculation can be found in the supplementary material (Section 3). The results are presented in Table 1 and compared with the integrated experimentally observed oxygen consumption after 200 days. The results suggest that very low amounts of TSS are almost completely mineralized within 200 days. The ratio of potential and observed oxygen consumption decreases with increasing algae load. After 200 days, only 69% of 80 g/m^2^ seem to be mineralized. The higher amount of algae mass might form a denser clogging layer that diminish homogenous flow, mass transfer and in this way microbial mineralization.

**Table 1:** Potential (pot) and experimentally (real) observed oxygen consumption of algae (quantified as total suspended solids: TSS) after 200 days

Algae load (g TSS/m^2^)	Algae mass (g TSS)	ΔO_2__,__pot_ (g)	ΔO_2,real,200d_ (g)	ΔO_2,pot/real_ (%)
20	0.08	0.11	0.11	100
40	0.16	0.21	0.16	76
80	0.32	0.42	0.29	69

**Fig. 3:**
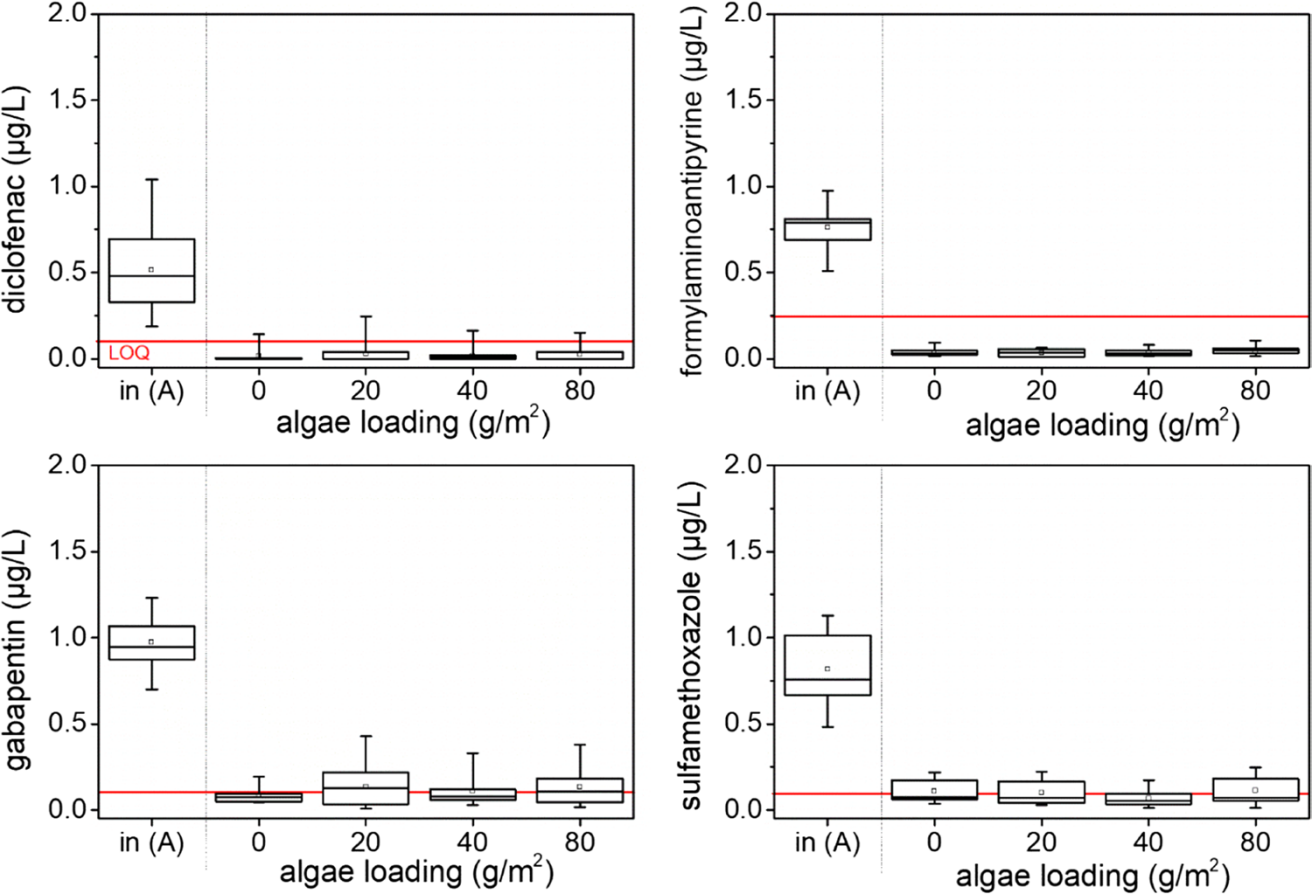
Influent (in A) and effluent concentrations of TOrCs for different algae loadings (expressed in g/m^2^ TSS) during the first 80 days after algae addition. The limit of quantification (LOQ) is indicated by a red line

**Fig. 4:**
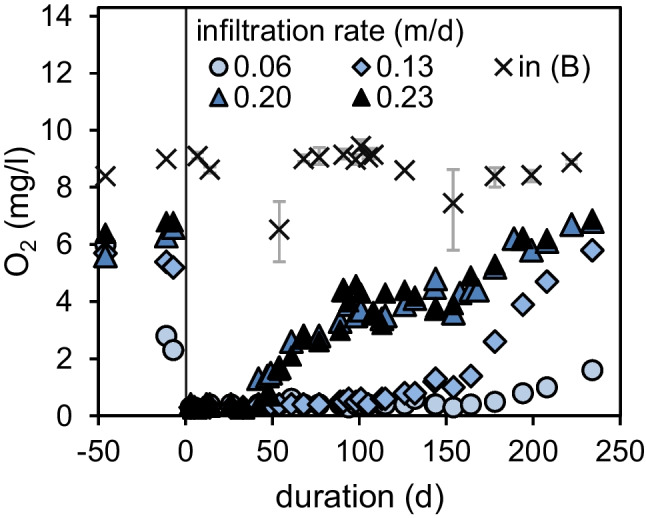
Dissolved oxygen concentrations in the influent and column effluents of series B before and after the addition of algae at day 0

### Impact of algae mass on DOM leaching

The sand columns of column series A with low TSS amounts (≤ 80 g/m^2^) and an infiltration rate of 0.38 m/d showed an average DOC removal of 4–7%, corresponding to an abatement of the UV_254_ absorption of 6 % (Figure S1). This indicates a slight removal of organic constituents, which contain aromatic structures and double bonds. The hardly degradable compounds such as humic substances can lead to a specialized microbial community that grows on refractory carbon substrates and thus promote TOrCs removal (Li et al. 2013; Rauch-Williams et al. 2010). A washout of DOC occurred in columns with the highest algae loading of 160 g/m^2^ during the first ten days. No long-term release of DOC from deposited algae was observed at infiltration rates exceeding 0.23 m/d.

**Fig. 5:**
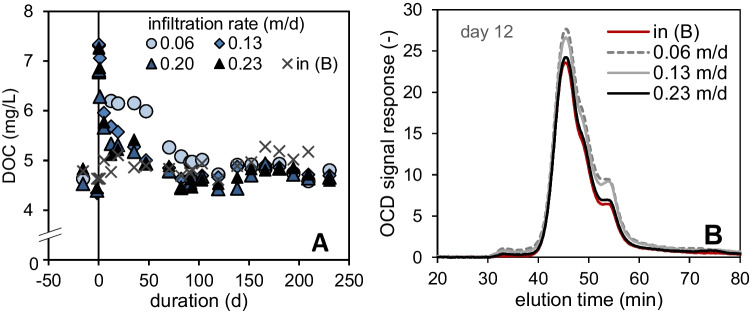
(**A**) DOC concentrations in column influents (in (B)) and effluents and (**B**) LC-OCD-chromatograms for column influent and column effluents 12 days after algae addition. The peaks of the DOC fractionated by size exclusion appear as follows: biopolymers (~32 min), humic substances (~42 min), and low molecular weight acids at (~53 min)

### Impact of algae mass on TOrCs removal

After the initial phase of more than 150 days, all sand columns revealed a removal of formylaminoantipyrine, sulfamethoxazole, gabapentin, and diclofenac before algae addition. The addition of 20–80 g/m^2^ did not affect the removal, and effluent concentration was still close to the limit of quantification (LOQ) of 0.1 µg/L for diclofenac, gabapentin, and sulfamethoxazole and below 0.25 µg/L for formylaminoantipyrine, respectively (Fig. 3). The relative concentration over the experimental runtime can be found in the supporting information (Figure S2). Sorption to algal biomass can lead to decreased TOrCs effluent concentrations. However, Wilt et al. (2016) report less than 20 % of the removal of carbamazepine and diclofenac due to sorption onto micro-algae in batch experiments.

Contrary to the hypothesis of better TOrCs removal under BDOC limited conditions (Hellauer et al. 2017a; Regnery et al. 2016), the availability of easily degradable BDOC from algae mass (≤ 80 g/m^2^ TSS) showed no enduring negative influence on TOrCs removals for the investigated column length. This observation corresponds with other laboratory sand column tests of Onesios and Bouwer (2012), where the removal of the majority of tested TOrCs did not show a dependency on added acetate concentrations. However, in their tests, gabapentin and diclofenac were recalcitrant. Zhang et al. (2019) observed an enhanced removal of diclofenac when increasing the BDOC with acetate addition. In another study, the removal efficiencies of acidic TOrCs like gabapentin and diclofenac decreased under limited BDOC conditions (Maeng et al. 2011). A correlation of the varying TOrC removal in the different studies with DOC removal can be found in the review of Filter et al. (2021).

Carbamazepine and benzotriazole were not removed in all columns and behaved recalcitrantly during the whole experiment. The respective data is shown in the supplementary material (Figure S3 and S4). Especially carbamazepine is considered as hardly biodegradable and its removal in biological filter systems can be often attributed to sorption (Jekel et al. 2015). The removal of benzotriazole in such systems is reported to be inconsistent (Filter et al. 2021).

### Impact of flow rate on oxygen consumption

Since the infiltration rate affects the oxygen consumption and thus the TOrCs removal, infiltration rates were varied at high algae loadings of 160 g TSS/m^2^. However, the infiltration rate highly depends on the amount of algal mass as algae provoke clogging and probably formation of preferential flow paths. Naghavi and Malone (1986) investigated the head-loss in fine sand filters caused by algae for different grain sizes (0.06–0.2 mm). They found a severe clogging at already 20–40 g TSS/m^2^ for high infiltration rates of 226 m/d. Furthermore, they reported an enhanced clogging for smaller grain sizes. In one column of series B, the intended infiltration rate of 0.4 m/d (for comparison with series A) had to be reduced to 0.23 m/d due to reduced permeability and thus two columns were operated similarly with 0.20 and 0.23 m/d, showing a comparable behavior in oxygen consumption, DOC release, and TOrCs removal.

During the pre-phase of series B, a dissolved oxygen consumption of around 2 mg/L was observed at an infiltration rate of 0.36 m/d for all columns (Fig. 4). The adjustment of flow rates just before algae addition led to higher oxygen consumptions for lower flow rates (0.06 and 0.13 m/d). After the addition of algae at day 0, oxygen was consumed completely in all columns for approximately 40 days. Thereafter, oxygen was detected in the column effluents with the highest infiltration rates of 0.20 and 0.23 m/d. 140 days after algae addition, oxygen occurred also in the effluent at an infiltration rate of 0.13 m/d.

### Impact of flow rate on DOM leaching

Low infiltration rates of 0.06 and 0.13 m/d caused increased effluent concentrations of biopolymers, humic substances, and low molecular weight acids (Fig. 5). Possibly the released DOC is less diluted and assimilated to a lower extent in biofilms in the absence of residual oxygen. Due to anoxic conditions, decay of organic compounds might have caused a release of low molecular weight acids, visible at a retention time of 53 minutes in the LC-OCD chromatogram (Fig. 5B). However, easily biodegradable DOC compounds might have been already degraded in the sand layer.

### Impact of flow rate on TOrCs elimination

**Fig. 6:**
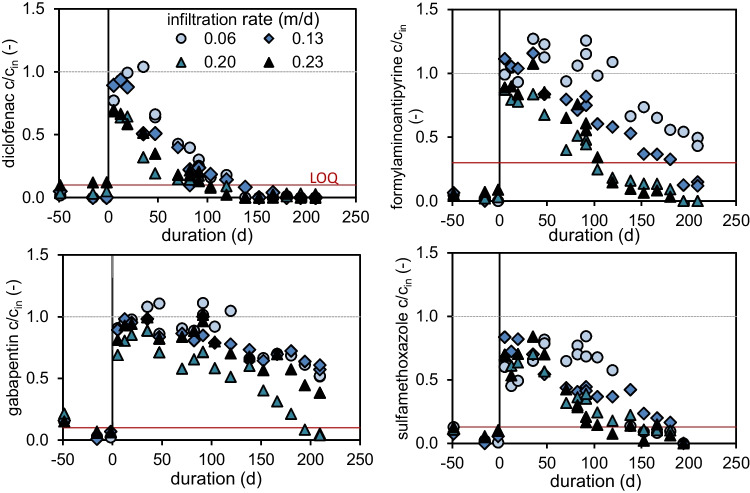
Relative effluent concentrations of diclofenac (*c*_in_: 0.97 ± 0.14 µg/L), formylaminonatipyrine (*c*_in_: 0.79 ± 0.09 µg/L), gabapentin (*c*_in_: 1.01 ± 0.11 µg/L), and sulfamethoxazole (*c*_in_: 0.75 ± 0.18 µg/L) for columns fed at different infiltrations rates before and after receiving 160 g/m^2^ algae TSS

A high algae loading of 160 g/m^2^ and infiltration rates of 0.23 m/d or even lower caused a complete oxygen depletion as discussed previously (Fig. 4). Accordingly, TOrC removals were significantly inhibited after the addition of algae (Fig. 6). Relative concentrations exceeding 1 most likely occurred due inaccuracies in TOrC quantification. However, higher effluent than influent concentration can also be caused by reversible sorption processes or backwards transformation of transformation products in the influent to the analyzed compound.

DO is one of the key parameters for MAR operation since it strongly determines the removal of organic compounds. Furthermore, a variety of TOrCs can be biologically transformed under oxic conditions (Regnery et al. 2017), whereas it can be severely inhibited under anoxic conditions.

Diclofenac elimination increased shortly after TSS addition almost independently from infiltration rates. As diclofenac removal is suspected to be co-metabolic (Zhang et al. 2019), the addition of primary substrate possibly increased the heterotrophic bioactivity that catalyzed diclofenac transformation. Several transformation pathways were identified for gabapentin under oxic conditions by Henning et al. (2018). After algae addition, gabapentin removal recovered only slowly, even after the column effluents reached oxic conditions again. This observation indicates a high sensitivity of gabapentin degrading microbiome regarding a sudden addition of organic carbon and the resulting anoxic conditions due to oxygen consumption. The recovery of the gabapentin removal did not show a clear dependency on the infiltration rate. However, increased infiltration rates of 0.20 and 0.23 m/d indicated a faster recovery.

Formylaminoantipyrine removal showed a clear dependency on infiltration rate and related oxygen supply.

A comparable behavior was observed for sulfamethoxazole, which is considered to be degradable under oxic as well as anaerobic but not under anoxic conditions (Baumgarten et al. 2011). The removal decreased by about 50–80% after algae addition. The recovery of sulfamethoxazole degradation is slower compared to diclofenac and depends on the infiltration rate, but not as pronounced as for formylaminoantipyrine. This is indicating a sensitive microbiom, catalyzing sulfamethoxazole transformation. That concurs with the reported long adaptation times, needed to establish sulfamethoxazole removal in sand column systems (Baumgarten et al. 2011).

## Conclusions

Low algae depositions at high infiltration rates have no impact on the removal of the investigated TOrCs if the sand filter remains oxic. Anoxic conditions caused by oxygen depletion due to deposited algae completely inhibited the removal of diclofenac, formylaminoantipyrine, gabapentin, and sulfamethoxazole. The recovery of the TOrC removals indicates a differing resilience of the microbiom catalyzing the transformation of the respective TOrCs. There was no evidence that the introduction of an additional organic substrate had lasting impacts the TOrCs removal, as long as oxic conditions were retained.

Decreasing the influent BDOC to enhance TOrCs removal cannot be the only focus for MAR treatment design. Even if the influent water is oxic and has a low BDOC concentration, the nutrient content of phosphate and nitrate can lead to formation of algae which, when filtered, can significantly consume oxygen and might disturb TOrCs transformation processes in the subsequent sand layers.

Besides nutrient control in the surface water or respective infiltration basins, protection against light by covering the sand filter could be another effective way to prevent intensive algal growth and sustain an increased oxic infiltration zone. This suggestion is supported by Campos et al. (2002) who investigated the biomass development in slow sand filters. They found 10 times less biomass in a covered infiltration basin compared to an open filter. The decreased biomass amount is also reflected by a decreased oxygen consumption in covered sand filters (Huisman and Wood 1974).

Another possibility to minimize the input of microalgae into SAT infiltration basins could be preceding micro-sieving of the applied surface water. Furthermore, it is recommended to consider regeneration of blocked SAT basins at an early stage to sustain infiltration rates and minimize oxygen consumption by accumulated particulate biomass.

## Supplementary information

Below is the link to the electronic supplementary material.

Supplementary file1 (DOCX 2410 KB)

## Data Availability

The data discussed in the manuscript are included in the text itself or in the supplementary material.
