# Supplementary material for: Impacts of autochthonous particulate organic matter on redox-conditions and elimination of trace organic chemicals in managed aquifer recharge
Source: Environ Sci Pollut Res Int. 2023 Jan 23;30(15):44121–9. doi: 10.1007/s11356-023-25286-0 (PMC10076367; doi:10.1007/s11356-023-25286-0)
Supplement: Supplementary file 1 — Supplementary file1 (DOCX 2410 KB) [file 11356_2023_25286_MOESM1_ESM.docx]

Supplementary Material

Impacts of autochthonous particulate organic matter on redox-conditions and elimination of trace organic chemicals in managed aquifer recharge

Josefine Filter^a,^*, Till Ermisch^a^, Aki Sebastian Ruhl^a,b^, Martin Jekel^a^

^a^Technische Universität Berlin, Chair of Water Quality Control, KF4, Straße des 17. Juni 135, 10623, Berlin, Germany

^b^ German Environment Agency, Section II 3.3, Schichauweg 58, 12307 Berlin, Germany

*Corresponding author. Tel.: +49 30 314 25367, E-mail address: [josefine.filter@tu-berlin.de](mailto:josefine.filter@tu-berlin.de)

Content

[1. Checking the practical significance of an area load of 80 g/m^2^ dry algae mass 2](#_Toc119779158)

[2. Calculation of the Hydraulic Retention Time (HRT) 2](#_Toc119779159)

[3. Calculation of potential oxygen demand 3](#_Toc119779160)

[4. DOC and UV_254_ in influents and effluents for different algae loadings (series A) 3](#_Toc119779161)

[5. TOrC Concentration over experimental runtime in column system A 3](#_Toc119779162)

[6. Concentrations and removals of carbamazepine and benzotriazole 4](#_Toc119779163)

[References 5](#_Toc119779164)

# Checking the practical significance of an area load of 80 g/m^2^ dry algae mass

In the following the theoretical run time of a MAR system is calculated until 80 g/m^2^ dry algae mass would accumulate. The raw water is considered as mesotrophic/ eutrophic with:

- a Phosphorous concentration of 0.02 mg/L (Chapra, 1997)

⬄ corresponding to 0.42 mg/L carbon according to the Redfield Ratio

⬄ and 25 µg/L Chlorophyll a (Chla) assuming 30 µgChla per mgC (range in Chapra (1997): 10-50 µgChla per mgC)

The MAR-System is loaded with an infiltration rate of 0.4 m^3^/(m^2^d) which results in an area load (C_load_) of 0.168 g/(m^2^d) C.

Assuming a carbon decay rate constant of 0.3 1/d (range in Reynolds (2006): 0.1-0.5 1/d) the carbon area is decreassed by the loss of carbon in one day:

$$C_{decay}=C_{load}-(C_{load}\cdot e^{-k\cdot\boldsymbol{t}})=0.336\frac{gC}{m^{2}\cdot d}-\left( 0.336\frac{gC}{m^{2}\cdot d}\cdot e^{-0.03\frac{1}{d}\cdot\boldsymbol{1}\boldsymbol{d}} \right)=0.01\frac{gC}{m^{2}\cdot d}$$

=> Netto area load: $C_{load,netto}=C_{load}-C_{decay}=0.336\frac{gC}{m^{2}\cdot d}-0.01\frac{gC}{m^{2}\cdot d}=0.326\frac{gC}{m^{2}\cdot d}$

Assuming a carbon content of 0.5 g C per g TSS the theoretical TSS load can be calculated the following:

${TSS}_{load, theor}=(C_{load,netto}\cdot\boldsymbol{t})/0.5\frac{gC}{gTSS}$

The time until the theoretical TSS load reaches the applied TSS load of 80 g/m^2^ was solved numerical with *EXCEL solver* by varying **t** to minimize${{(TSS}_{load}-{TSS}_{load,theor})}^{2}$.

The calculated run time for MAR basin are 123 days until 80 g TSS per m^2^ are accumulated for the assumed raw water characteristics.

# Calculation of the Hydraulic Retention Time (HRT)

| infiltration velocity (m/d) | Loading Rate LR (mL/h) | Bed volume BV (L) | Assumed porosity  *p* ^(1)^ | Pore volume PV (L) | HRT^(2)^ (h) |
| --- | --- | --- | --- | --- | --- |
| 0.40 | 50 | 1.2 | 0.4 | 0.48 | 10 |
| 0.23 | 29 | 1.0 |  | 0.40 | 14 |
| 0.13 | 18 | 1.0 |  | 0.40 | 22 |
| 0.06 | 8 | 1.0 |  | 0.40 | 50 |

1. porosity of sand sediments: 0.3-0.4 (Todd and Mays, 2005)
2. $HRT=\frac{PV}{LR}=\frac{BV\cdot p}{LR}$

# Calculation of potential oxygen demand

- based on the following assumption of 1 mol C_org_ with an oxidation level (mean oxidation number) of ±0 is mineralized with 1 mol O_2_ to 1 mol CO_2_: $1C_{org}+1O_{2}=1{CO}_{2}$

${\Delta O}_{2,pot}=\frac{M_{O2}}{M_{C,org}}=\frac{32\frac{g}{mol}}{12\frac{g}{mol}}=2.7$ => the molar mass ratio for oxidizing 1 mol C_org_

# DOC and UV_254_ in influents and effluents for different algae loadings (series A)


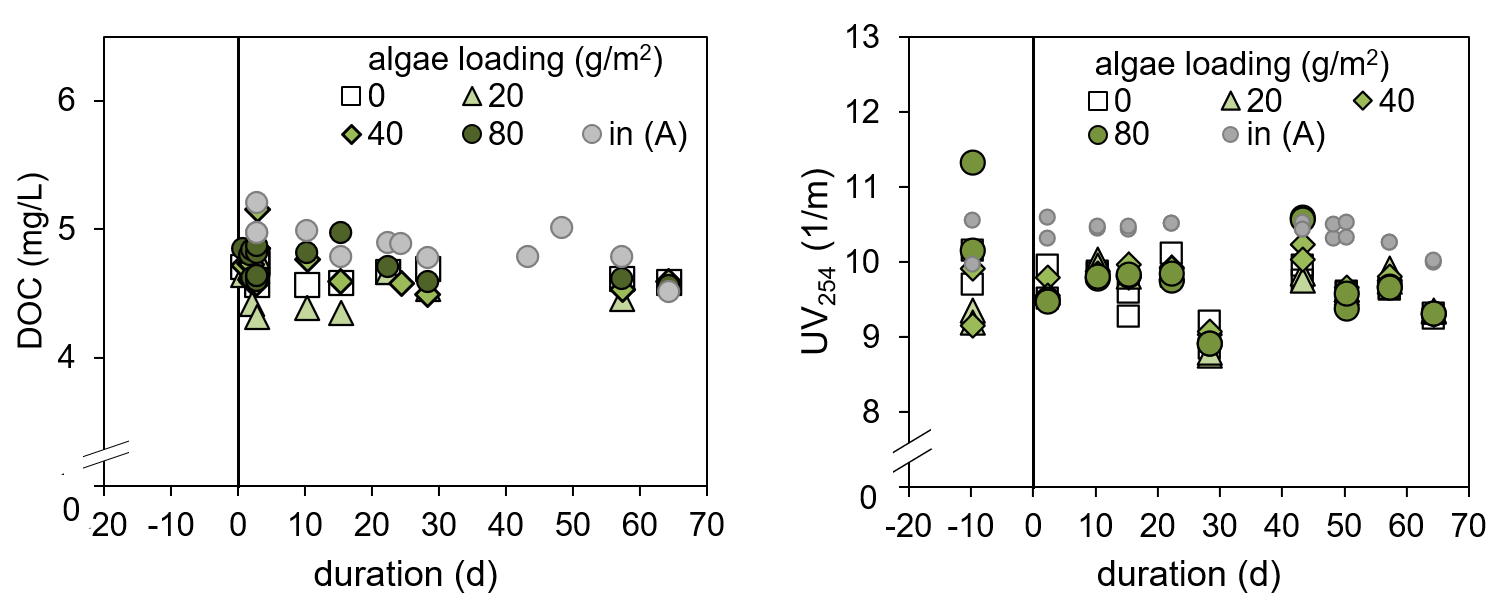


Figure S1: DOC concentrations and UV absorptions (254 nm) in column effluents and influents of series A 10 days before algae loading and during the first 70 days after algae addition

# TOrC concentration over experimental runtime in column system A


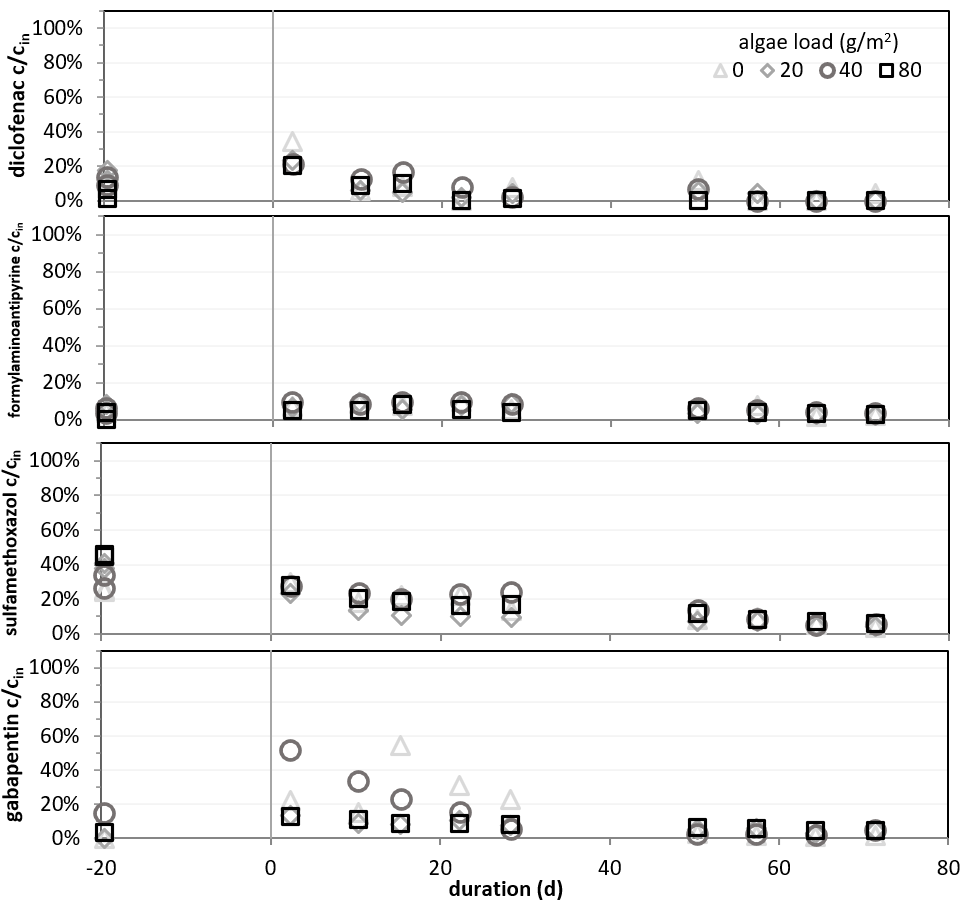


Figure S2: Relative effluent concentrations of diclofenac, formylaminonatipyrine, gabapentin and sulfamethoxazole before and after receiving algae TSS

# Concentrations and removals of carbamazepine and benzotriazole





Figure S3: Removal of carbamazepine and benzotriazole in dependency of the algae load


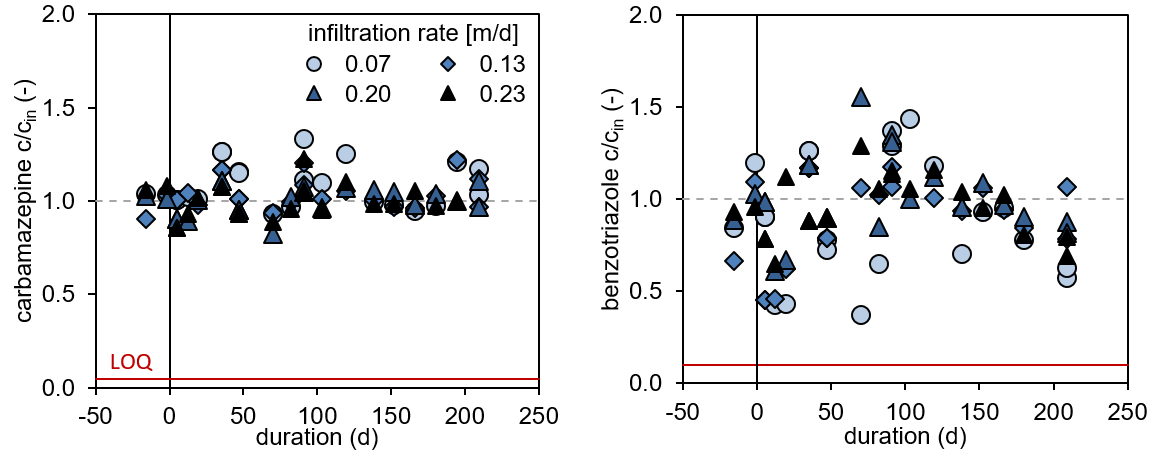


Figure S4: Removal of carbamazepine and benzotriazole with varying infiltration velocities

References

Chapra, S.C., 1997. Surface water-quality modeling: Chapra, Steven, C. McGraw-Hill, New York.

Reynolds, C.S., 2006. Ecology of phytoplankton. Ecology, biodiversity, and conservation. Cambridge University Press, Cambridge.

Todd, D.K., Mays, L.W., 2005. Groundwater hydrology, 3rd ed. ed. John Wiley & Sons, Hoboken (Nueva Jersey, Estados Unidos).
